# Tumor Hypoxia: Impact on Radiation Therapy and Molecular Pathways

**DOI:** 10.3389/fonc.2020.00562

**Published:** 2020-04-21

**Authors:** Brita Singers Sørensen, Michael R. Horsman

**Affiliations:** Experimental Clinical Oncology—Department of Oncology, Aarhus University Hospital, Aarhus, Denmark

**Keywords:** hypoxia, radiation response, gene regulation, intracellular signaling, hypoxia classifyer

## Abstract

Tumor hypoxia is a common feature of the microenvironment in solid tumors, primarily due to an inadequate, and heterogeneous vascular network. It is associated with resistance to radiotherapy and results in a poorer clinical outcome. The presence of hypoxia in tumors can be identified by various invasive and non-invasive techniques, and there are a number of approaches by which hypoxia can be modified to improve outcome. However, despite these factors and the ongoing extensive pre-clinical studies, the clinical focus on hypoxia is still to a large extent lacking. Hypoxia is a major cellular stress factor and affects a wide range of molecular pathways, and further understanding of the molecular processes involved may lead to greater clinical applicability of hypoxic modifiers. This review is a discussion of the characteristics of tumor hypoxia, hypoxia-related molecular pathways, and the role of hypoxia in treatment resistance. Understanding the molecular aspects of hypoxia will improve our ability to clinically monitor hypoxia and to predict and modify the therapeutic response.

## Characteristics of Tumor Hypoxia

**Figure 1:**
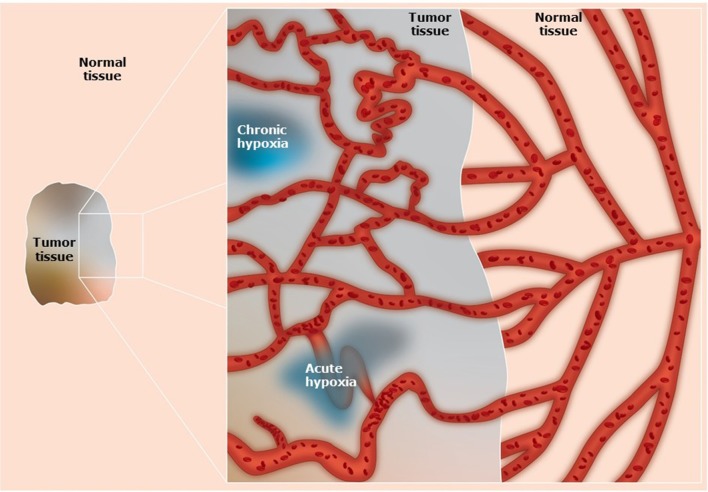
Schematic illustration of the vascular networks in tumors and associated normal tissues. Compared to the well-organized blood supply of normal tissues, in tumors the system is primitive and chaotic. The tumor vascular supply shows abnormal vascular density, contour irregularities, enlarged vessels, vessels with blind ends, and transiently blocked vessels. In addition, there is a loss of hierarchy, a lack of regulatory control mechanisms, and the vessel walls can be structurally defective causing increased vascular permeability. These factors result in the development of diffusion limited chronic hypoxia and perfusion limited acute hypoxia.

Normal tissues require a regular supply of oxygen and nutrients to maintain viability, and a means for eliminating the waste products of metabolism (1, 2). These processes are achieved through a functional blood supply. Most solid tumors have the same metabolic requirements and to achieve this tumors initially utilize the blood supply of the host organ in which the tumor arises. Eventually that supply becomes inadequate in meeting the demands of the growing tumor mass (1, 2). To compensate, tumors develop their own functional vascular supply from the normal host vascular network by the process of angiogenesis (3, 4). However, despite the significance of this tumor neo-vasculature, the system formed is chaotic and primitive, suffering from numerous structural, and functional abnormalities (1, 2) (Figure 1). Consequently, it is actually unable to meet the metabolic demands of the developing tumor. Micro-regional areas are thus formed within the tumor that are characterized by glucose and energy deprivation, high lactate levels and extracellular acidity, and oxygen deficiency (1, 2).

The most extensively studied micro-environmental parameter is hypoxia. Hypoxia is a characteristic feature of most solid tumors and is generally defined as a state of reduced oxygenation that influences biological function (5). As such, it is usually considered as a single entity, which it most definitely is not. From histological data from patients with carcinoma of the bronchus, Thomlinson & Gray suggested that hypoxia could be present in tumors due to a diffusion limit of oxygen (6). Such hypoxia would be chronic in nature (Figure 1). Later it was proposed (7) and demonstrated (8) that a form of acute/transient hypoxia could occur, resulting from periodic fluctuations in blood flow (9) (Figure 1). This acute hypoxia can result from a complete shut-down in tumor blood flow thus causing ischemic hypoxia, or from a partial shut-down sufficient to induce hypoxia by preventing red blood cell flow yet allowing plasma flow to continue to supply nutrients. The cause of chronic hypoxia can also be multi-factorial. Although the result of a diffusion limitation, the actual distance from the blood vessel can be highly variable due to several factors. These include the oxygen carrying capacity of the blood, which can be “normal” or reduced as in anemic patients or smokers and the ability of hemoglobin to release oxygen (10). It also involves the intravascular oxygen partial pressure gradient (from the arterial to the venous end of the micro-vessels), and the level of oxygen consumption by the tumor cells and the tumor growth fraction, both of which can vary within and between tumors (10). One also has to consider the degree of oxygenation, which can vary from reasonably well-oxygenated through intermediate levels of hypoxia to severely hypoxic (11).

## Hypoxia and the Hypoxia-Inducible Factor (HIF) Regulatory Pathway

**Figure 2:**
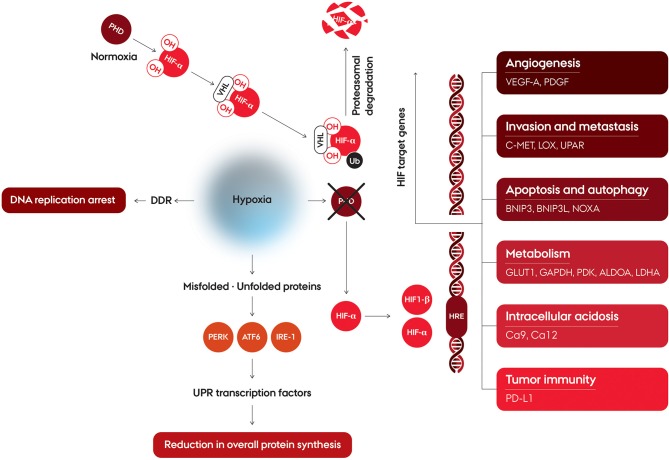
Schematic illustration of cellular pathways affected by hypoxia. Hypoxia affects regulation of hypoxia-inducible factors and induction of HIF target. HIF is a heterodimer consisting of an alpha (α) subunit (HIF-1α, HIF-2α or HIF-3α) and a HIF-1β subunit. Under normoxic conditions, HIF-α is rapidly degraded due to hydroxylation by prolyl hydroxylase domain (PHD) protein. The proline-hydroxylated HIF-α interacts with the von Hippel-Lindau protein (VHL), which targets HIF-α for ubiquitination and degradation via the proteasome. Under hypoxia, HIF-α is stable, and forms the active transcription complex with HIF-1β. After translocation to the nucleus the HIF heterodimer binds at the hypoxia response element (HRE) of target genes thereby initiating the transcription of the HIF target genes. At severe hypoxia, the cellular response also affects the DNA Damage Response (DDR), which leads to DNA replication arrest. Exposure to very low oxygen concentrations also leads to an reduction in mRNA translation initiation and overall protein synthesis, through an activation of the Untranslated Protein Response (UPR).

Hypoxia is a major cellular stress factor and in response to this condition, cells undergo a wide range of molecular changes. A number of cellular pathways are affected, including increased glycolysis, decrease of cell proliferation, and enhancement processes involved in angiogenesis and erythropoiesis (Figure 2). The hypoxia-mediated intracellular signaling pathways are pre-dominantly orchestrated by intracellular signaling, mainly under control by a family of transcription factors, the hypoxia inducible factors (HIFs) (12, 13). HIF upregulates target gene expression through binding at the hypoxia responsive elements (HREs) in the enhancer and promotor regions of the target genes (14). HIF binds to the DNA as a heterodimer consisting of a alpha (α) subunit (HIF-1α, HIF-2α, or HIF-3α) and a HIF-1β subunit (15). HIF-1β is constitutively expressed, while regulation of HIFα is controlled by tissue oxygenation status, through hydroxylation of two proline residues by prolyl hydroxylase domain proteins (PHD) 1-3 (16, 17) (Figure 2). Hydroxylation, occurring only in the presence of oxygen, promotes interaction with the von Hippel-Lindau tumor suppressor protein (pVHL), which targets HIFα for ubiquitination and subsequent proteasomal degradation (18, 19). At oxygen concentrations around 2% O_2_ and below this hydroxylation is suppressed leading HIFα to not be degraded (20, 21), and form the active transcription complex with HIF-1β, which induce transcriptional upregulation of a broad range of target genes (22–24). The regulation of HIF-α is not only affected by PHD1-3, since a large plethora of kinases are also involved in the regulation, either directly, or indirectly (15). The major HIF complexes are comprised of HIF-1β, and one of either HIF-1α or HIF-2α, which constitutes the transcription factors referred to as HIF1 and HIF2 (25). HIF1 and HIF2 have structural similarities and identical DNA recognition motifs, but binds to different cell-specific sites across the genome (26, 27). HIF-3α has a structural difference, in that it lacks the C-terminal TAD, and as such is not able to induce the expression of hypoxia-inducible target genes to the same extent as HIF-1α and HIF-2α. HIF-3α competes with HIF-1α or HIF-2α to bind HIF-1β, and can thereby act as a suppressor of HIF-dependent gene expression (25, 28). The HIFs have been shown to influence a large range of cellular functions (Figure 2), such as angiogenesis, invasion and metastasis, apoptosis and autophagy, metabolism, intracellular acidosis, and tumor immunity.

## Hypoxia and Cellular Stress Responses

Cancer cells adapt to hypoxia by a number of stress responses, mediated by the intracellular signaling aimed at facilitating the cells ability to cope with the microenvironment, and to alter the energy requirements as necessary. One of the stress responses is the unfolded protein response (UPR) activated in response to ER stress, endoplasmic reticulum stress, and leads to a downstream activation of adaptive mechanisms. ER stress is the result of an accumulation of unfolded or misfolded proteins, as oxygen depletion can interfere with protein folding (29, 30) Unlike HIF, which is activated at oxygen concentrations below 2%, UPR is activated at exposure to more severe hypoxia (<0.02% O_2_) (31). The UPR is a complex of intracellular signaling pathways which are mediated by three independent ER transmembrane proteins: PKR-like ER kinase (PERK), Activating Transcription Factor 6 (ATF6) and inositol-requiring enzyme 1(IRE-1) (32). Exposure to severe hypoxia leads to a reduction in mRNA translation initiation and overall protein synthesis, through a activation of PERK which subsequently phosphorylates eIF2α (33) (Figure 2). Activated ATF6 and IRE-11 directly modulates transcriptional induction of UPR target genes. Activation of IRE-1 leads to expression of a panel of genes maintaining metabolic homeostasis and ER through activation of a transcription factor, spliced XBP1 (XBP1s) (34–36). ATF6 is cleaved in the Golgi apparatus, where after the active transcriptional form, ATF6f, translocate to the nucleus and induce transcription of the UPR target genes (29, 37, 38).

Translation of the majority of genes is inhibited under these conditions, but due to regulatory sequences in the 5′ untranslated regions, some gene transcripts are able to escape this inhibition, resulting in an alteration in differential protein expression during hypoxia due to the change in translational efficiency (33, 39).

UPR has been suggested to induce autophagy, an intracellular self-degradation process which can both induce or protect from cell death, through the PERK and BNIP3 pathways (40, 41). The impact of hypoxia on autophagy pathways in malignant cells, and the balance of autophagy in survival and death pathways under hypoxia has shown to be complex. It is susceptible to the genetic background of the cells, as well as the severity of the oxygen deprivation and of other tumor microenvironmental factors (40, 41).

The cellular response to hypoxia also affects the DNA Damage Response (DDR) at very low oxygen concentrations, which includes DNA replication arrest and rapid accumulation of replication stress (Figure 2). This is thought to be due to the enzyme responsible for nucleotide production, ribonucleotide reductase, being dependent on cellular oxygen for its function and, therefore, compromised in hypoxic conditions (32, 42). The DDR involves a complex collaboration between signaling pathways activated due to different types of DNA damaging stresses, and the hypoxia induced effects includes both ATR- and ATM-mediated signaling, despite the absence of detectable DNA damage. This results in cellular protection of the replication forks, minimizing the risk of further genomic instability (42–44). Activation of p53 is a consequence of the hypoxia induced DDR, by phosphorylation at a number of residues (45, 46).

The tumor microenvironment is characterized by factors other than tumor hypoxia, such as low pH. Lactic acid accumulation can cause acidosis in solid tumors. In order to compensate for reduced mitochondrial ATP, low oxygen concentrations leads to anaerobic energy production and the formation of lactic acid production, referred to as the Pasteur effect (47). Significant disparities in the temporal and spatial distribution of areas in tumors with low oxygenation level and high level of acidosis results from tumor cells maintaining a high rate of glycolysis even in the presence of oxygen, a which is referred to as aerobic glycolysis or the Warburg effect (48–50). The cellular response in terms of DNA repair and gene transcription and translation succeeding combination of low oxygen concentration and low extracellular pH in combination has shown to be very different compared to the response to either hypoxia or acidosis alone (51, 52). While both hypoxia and acidosis greatly effects the cellular response, simultaneous hypoxia and acidosis *in vitro* suppresses metabolic rate and protein synthesis to a greater extent than each of the factors on their own (53).

## Immune Inflammatory Pathways

Cancer immunotherapy has resulted in unprecedented improvements in outcome in patients with a spectrum of solid tumors, and has established itself as the fourth modality in cancer treatment. This is primarily the result of development of vaccines and agents targeting immune regulatory checkpoints, namely the cytotoxic T-lymphocyte-associated protein 4 (CTLA-4), or programmed death 1 (PD-1) and programmed death 1 ligand (PD-L1) (54). Despite positive results, many patients show little or no response to vaccines and checkpoint inhibitors (55). The immune response to tumors is a complex balance between antitumor mechanisms, where infiltrating lymphocytes recognize tumor specific antigens on the surface of cancer cells and eliminate the cancer cells thereby decrease tumor growth, and the protumor inflammatory response, which increases immune tolerance, cell survival, and proliferation (56–58). There is evidence that radiation alone can induce an innate immune response, and recent studies have shown that the combination of radiotherapy with immunotherapy has the potential to be an effective treatment modality (59–61).

Hypoxia seems to play a significant role in influencing anti-cancer immune responses (62, 63). It promotes an immunosuppressive microenvironment by regulating the recruitment of T-cells, myeloid-derived suppressor cells (MDSCs), macrophages, and neutrophils (64, 65). In addition, hypoxia can have a negative effect on immunogenicity by altering the function of immune cells and/or increasing resistance of tumor cells to the cytolytic activity of immune effectors (66, 67). There is also evidence that hypoxia can influence immune checkpoints. A rapid and selective up-regulation of PD-L1 is induced by hypoxia on MDSCs, and significant increased expression of PD-L1 on macrophages, dendritic cells and tumor cells, all due to HIF1 binding directly to the HRE in the PD-L1 proximal promoter (68). Hypoxia has also been shown to regulate the CTLA-4 receptor, again potentially via HIF1 (69). Apart from direct immune suppressive effects, hypoxia can also indirectly affect immune response since it causes an increased accumulation of adenosine, drives the expression of vascular endothelial growth factor, and is associated with higher levels of lactate, all of which can inhibit anti-tumor immunity (62, 70). Interestingly, one pre-clinical study using a variety of tumor models showed that by allowing tumor-bearing mice to breathe high oxygen content gas (60% oxygen) rather than the normal 21% oxygen, resulted in an inhibition of tumor progression, a decrease in metastatic disease, and prolonged animal survival (67). This hyperoxia decreased tumor hypoxia, increased pro-inflammatory cytokines, decreased the levels of immunosuppressive molecules, and weakened immunosuppression by regulatory T-cells.

Clearly, there is a need to investigate role of hypoxia on immune response and understand how modifiers of hypoxia influence that response. Non-invasive imaging may be helpful in this context. Substantial pre-clinical and clinical effort has been made in finding clinically relevant approaches that can non-invasively identify hypoxia in tumors (71). The techniques include positron emission tomography (PET), magnetic resonance imaging, and computed tomography. Using these techniques, especially the PET-based approaches, one not only identifies tumor hypoxia, but also shows its relationship to patient outcome following radiotherapy (71). More recently, a PET based approach has also been developed for non-invasively imaging immunotherapy. It involves radiolabeling various monoclonal antibodies with 89-Zirconium (^89^Zr). Pre-clinically, these conjugates have included CD4 and CD8 antibodies (72), or an anti-PD-L1 antibody (73). Both approaches allowed for whole body visualization and evaluation of tumor response. Such approaches have even undergone clinical evaluation using ^89^Zr-labeled atezolizumab, an antibody against PD-L1, and the images obtained in cancer patients was able to assess response to PD-L1 blockade (74). Combining PET-hypoxia markers with immunotherapy based PET markers should allow us to investigate the interaction between both parameters and how that influences patient outcome.

## Significance of Hypoxia for Radiation Response

Estimates of tumor hypoxia obtained using electrodes, exogenous marker expression, or the upregulation of endogenous hypoxia-associated molecules, have not only demonstrated hypoxia to be a common feature of animal solid tumors, human tumor xenografts and human cancers (49, 75), but also a major negative factor influencing tumor radiation response. Pre-clinical studies in the early 1950s demonstrated that when the partial pressure of oxygen was reduced below about 20 mmHg at the time of irradiation cells became resistant to the radiation damage (76). When radiation is absorbed in biological material, highly reactive free radicals are produced either directly or indirectly in the target. These radicals are unstable and will react with oxygen to change the chemical composition of the target, ultimately causing damage. However, under hypoxic conditions the target can be chemically restored to its original form. Typically, under hypoxia one requires 2.5–3.0 fold higher radiation doses to induce the same level of damage as seen under normoxic conditions (77). The type of hypoxia (i.e., chronic or acute) is irrelevant for the initial radioprotection. However, while chronically hypoxic cells are generally also nutrient deprived, acutely hypoxic cells are hypoxic for only a short period (78) and as such are less likely to be nutrient deprived, and this could play a role in influencing the cells ability to repair the radiation damage, thus making acute hypoxia a more resistant factor.

Regardless of whether one type of hypoxia is more of a negative factor, there is good clinical evidence that hypoxia significantly impacts patient outcome following radiation therapy (71). Consequently, substantial effort has been made in the last 50 years to identify approaches that can overcome hypoxia-induced radiation resistance (1, 71). These have involved using agents that either increase oxygen delivery, radiosensitize the hypoxic cells, or preferentially kill them. Attempts have also been made to use dose painting, whereby the hypoxic areas are identified and the radiation dose to these areas is increased, or the use of high LET (linear energy transfer) radiation where hypoxia is less of an issue (79). However, despite the pre-clinical and even clinical demonstrations of the benefit of several of these approaches, only one approach has become established in routine clinical practice and that is the hypoxic cell radiosensitizer nimorazole, and only in head & neck squamous cell carcinoma and only in Denmark (80) and Norway (81).

## Vascular Targeting Agents and Hypoxia

A principal factor controlling the tumor microenvironment, and thus the degree of hypoxia, is its vascular supply. As a result, any treatment that modifies this tumor vascular supply can consequently change the level of hypoxia. One such group are the so-called vascular targeting agents (VTAs). These include angiogenesis inhibitors (AIs) that inhibit the development of the tumor neo-vasculature, and vascular disrupting agents (VDAs) that damage the already establish tumor vascular supply (82, 83).

With VDAs, the vascular damage induced causes a reduction in tumor blood flow and this increases the adverse microenvironmental conditions within tumors leading to substantial cell killing and subsequent increase in necrosis (82, 84, 85). The overall result is a reduction in tumor volume. AIs also inhibit tumor growth, but their effects on the tumor vascular supply and microenvironment are more complex and somewhat controversial. Some years ago it was suggested that rather than AIs simply stopping the angiogenesis process and thus decreasing vessel density they could also actually reduce or abolish the vascular abnormalities of the remaining vessels, causing vessel stabilization resulting in a more efficient vasculature similar to that seen in normal tissues. This stabilization process was termed “normalization” (86) and the more stable, organized vasculature that resulted would likely lead to a better delivery of oxygen and nutrients to the tumor, thus reducing the degree of tumor hypoxia. Numerous studies have since reported that treatment with a range of AIs can indeed give rise to an apparent decrease in tumor hypoxia (82, 84). The first study that demonstrated an improvement in oxygenation status that was associated with vessel normalization was that of Winkler and colleagues (87), using the anti-VEGF (vascular endothelial growth factor) monoclonal antibody DC101. Using a human glioblastoma xenograft grown orthotopically in the mouse brain they found that during treatment with DC101 there was a significant decrease in the level of binding of the hypoxic marker, pimonidazole, and a similar increase in radiation sensitivity, an affect that was clearly associated with pericyte recruitment. They also found that when pericyte coverage was maximal there was an upregulation of human angiopoietin-1 (Ang-1) and Ephrin B2. Ang-1 is associated with pericyte recruitment and additional studies showed that an increased synthesis of Ang-1 mRNA resulted in an increased Ang-1 protein deposition close to its receptor Tie2 on the endothelial cells (87). Furthermore, when using a Tie2-blocking antibody or peptide to block Ang-1/Tie2 signaling, DC101 was unable to increase pericyte coverage of vessels. However, the reported improvements in oxygenation by AIs are not all due to vessel normalization. Using SU5416, an antagonist of the VEGF receptor, the increase in tumor oxygenation resulted from an inhibition of mitochondrial respiration, thereby decreasing hypoxia by increasing the oxygen diffusion distance (88).

Regardless of the mechanisms for these decreases in tumor hypoxia, the improved oxygenation in both these studies was somewhat transient and only lasted for a period of a few days despite the AI treatment being continued. This “narrow window” of improved oxygenation has also been seen with thalidomide (89, 90), a nucleolin antagonist (91), and bevacizumab (92–95), regardless of the technique used to monitor the changes in oxygenation/hypoxia. The transient nature of this effect would suggest that the timing of hypoxia measurement is critical. In fact, two studies reported both a decrease and increase in hypoxia depending on the time of measurement after treatment with either DC101 (96) or bevacizumab (95).

Although at least one clinical study suggested an apparent improvement in oxygenation with AI therapy (97), several pre-clinical studies reported no change in tumor oxygenation status despite the AIs causing a decrease in vascular density and blood perfusion (1, 98). More significantly, in the majority of reported pre-clinical studies these AI-induced anti-vascular effects actually led to an increase in hypoxia, in line with what one would expect (1, 82). It could be argued that these different effects on tumor oxygenation status could be the result of using different drugs, doses, scheduling, or the time of hypoxia assessment. However, it seems more likely that the effects are a tumor dependent phenomena. This is probably best illustrated using DC101, where one study showed that 2 days after treating animals with DC101 (3 × 40 mg/kg), U87 gliomas were significantly better oxygenated when measured using a hypoxic specific marker (87). Yet another study using the same drug, almost identical dose schedule (3 × 45 mg/kg), and similar hypoxic specific marker, found that 2 days after treatment, MCa4/MCa35 mammary carcinomas were significantly more hypoxic (99). This same controversy was seen in the limited clinical studies in which both a decrease (100) and an increase (101, 102) in tumor hypoxia have been reported. Such findings clearly argue against making sweeping statements about the effects of AIs on tumor hypoxia and that either measurements of the oxygenation status need to be routinely made when AIs are administered or that they be given in such a way as to avoid any negative influence on the conventional treatment with which they are combined.

## Molecular Hypoxia Biomarkers

To take advantage of the cellular response to hypoxia, the use of expression levels of hypoxia induced genes as biomarkers for tumor hypoxia has been widely investigated. Initially, single genes such as HIF-1, Ca9, and Glut1 measured either at the protein level, with for example immunohistochemistry, or on the mRNA level with for instance qPCR, was used in a range of studies (103–106). The use of single gene expression markers for tumor hypoxia has often led to conflicting reports, due to the genes being influenced by factors other than hypoxia, such as extracellular pH or glucose concentrations (107, 108). Ca9 expression was one such factor proposed as a hypoxia marker in a number of studies, however other experimental studies clearly demonstrated that hypoxia and Ca9 expression did not exclusively correlate (109). Certain microRNAs (miRNAs) have also demonstrated to be inducible by hypoxia (110, 111), as for example hsa-mir-210 which has shown to be hypoxia related and to have prognostic significance in several tumor types, e.g., in cervical cancer (112), in breast cancer (113), and in bladder cancer (114).

Progresses in gene expression profiling have let to a higher level of understanding of the biology of hypoxia, and development of hypoxic signatures based on a number of genes rather than on single genes as biomarkers for tumor hypoxia (115–122). These have typically been developed by determining global gene expression levels by gene expression arrays, and identifying genes preferentially upregulated by hypoxia based on either *in vitro* or clinically derived gene expression data sets. There is no consensus to the optimal way to develop gene expression signatures, and the currently published hypoxia gene expression signatures are at different stages in respect to clinical usability and validation (123).

The Toustrup 15-gene-classifier was developed from a panel of genes, identified in an *in vitro* study in a panel of Head and Neck Squamous Cell Carcinoma (HNSCC) cell lines as upregulated by low oxygen concentration, independent of pH. It was developed in a training cohort of 58 HNSCC patients with the oxygenation status measured using an oxygen electrode. The classifier was validated in the DAHANCA 5 cohort, which is a Danish study where patients were randomized to receive either the previous mentioned hypoxic cell radiosensitizer nimorazole, or placebo, with radiotherapy. The classifier was in this cohort demonstrated to be both prognostic and have predictive impact for hypoxic modification (124). The 26-gene classifier by Eustace et al. (121), is another hypoxia signature in HNSCC. This signature is based on a metagene signature developed for patients with breast, lung and head and neck cancers. In the Dutch ARCON trial, which compared treatment with radiotherapy combined with carbogen and nicotinamide, two hypoxia modifying agents, compared to radiotherapy alone in patients with laryngeal cancer, the patients classified as “more hypoxic” according to the 26-gene classifier showed a significantly improved locoregional control in when treated with the modifying agents (123, 125).

Several studies have aimed at comparing the published gene signatures (126–129), but with the constraint that common analyzing methods have been used, such as the two-class k-means clustering, and not the validated analysis method, which for some of the gene signatures include cutoff values.

To utilize the biological knowledge, studies have been focused on combining gene signatures for hypoxia with other factors known to affect cellular factors influencing the response to radiotherapy, such as markers for cancer stem cells (129), and for proliferation and DNA repair (119). Currently, for all signatures there is a need for a continued validation, both at the technical and clinical level (130, 131), especially to be able to advance from retrospective to prospective classification of the hypoxic status of patients and subsequently the assignment to hypoxia-modifying therapies in the clinic.

Tumor hypoxia mediates intercellular signaling through the regulation of many cytokines and angiogenic factors (CAF), and serum or plasma levels of hypoxia associated proteins have also been suggested as markers for hypoxia (132–134). One of the proteins which have been intensively studied is osteopontin (OPN). OPN has both *in vitro* and *in vivo* shown to be upregulated by hypoxia (108, 135), and clinical studies have found a high level of OPN to be associated with a poor prognosis, both in HNSCC (136, 137) and small cell lung cancer (138). The findings of a correlation of OPN levels and hypoxia is not consistent across studies (139), and it has been demonstrated that the measured level of OPN is sensitive to the choice of analysis platform (140). Nonetheless, hypoxia associated circulating proteins could add prognostic information on patient outcome.

## Conclusion

In the age of targeted therapies, hypoxia has to be considered the ultimate target. Hypoxia exists in virtually all solid tumor types, it influences patient response to radio-, chemo-, and immune-therapy, and plays a major role in malignant progression. Its presence in tumors can be identified by various invasive and non-invasive techniques, and there are a number of approaches by which hypoxia can be modified to improve outcome. However, despite these factors and the ongoing extensive pre-clinical studies, the clinical focus on hypoxia is still to a large extent lacking. Molecular pathways are the fundamental background for the cellular response to hypoxia, and further understanding of the molecular processes involved may help overcome this limitation.

## Author Contributions

MH and BS formulated the topic of the review and drafted and approved the manuscript.

## Conflict of Interest

BS was a co-inventor on a patent on a method (gene expression profile) for determining clinically relevant hypoxia in cancer (WO2012146259 A1) that is owned by Aarhus University, Aarhus, Denmark. The remaining author declares that the research was conducted in the absence of any commercial or financial relationships that could be construed as a potential conflict of interest.
